# OutlierNets: Highly Compact Deep Autoencoder Network Architectures for On-Device Acoustic Anomaly Detection

**DOI:** 10.3390/s21144805

**Published:** 2021-07-14

**Authors:** Saad Abbasi, Mahmoud Famouri, Mohammad Javad Shafiee, Alexander Wong

**Affiliations:** 1 Department of Systems Design Engineering, University of Waterloo, Waterloo, ON N2L 3G1, Canada; srabbasi@uwaterloo.ca (S.A.); mjshafiee@uwaterloo.ca (M.J.S.); 2 DarwinAI Corp., Waterloo, ON N2V 1K4, Canada; mahmoud@darwinai.ca; 3 Waterloo Artificial Intelligence Institute, Waterloo, ON N2L 3G1, Canada

**Keywords:** acoustic anomaly detection, embedded machine learning, deep learning, unsupervised learning

## Abstract

Human operators often diagnose industrial machinery via anomalous sounds. Given the new advances in the field of machine learning, automated acoustic anomaly detection can lead to reliable maintenance of machinery. However, deep learning-driven anomaly detection methods often require an extensive amount of computational resources prohibiting their deployment in factories. Here we explore a machine-driven design exploration strategy to create **OutlierNets**, a family of highly compact deep convolutional autoencoder network architectures featuring as few as **686** parameters, model sizes as small as **2.7 KB**, and as low as **2.8 million** FLOPs, with a detection accuracy matching or exceeding published architectures with as many as 4 million parameters. The architectures are deployed on an Intel Core i5 as well as a ARM Cortex A72 to assess performance on hardware that is likely to be used in industry. Experimental results on the model’s latency show that the OutlierNet architectures can achieve as much as **30× **lower latency than published networks.

## 1. Introduction

Acoustic anomalies are one of the primary ways through which malfunctioning machinery or industrial processes are monitored. However, this detection of abnormal sounds is typically done subjectively via human operators who need prior experience. This is an important problem as automatic detection of acoustic abnormalities can lead to more reliable predictive maintenance and potentially eliminate the need for manual monitoring. More importantly, interconnected intelligent monitoring systems are also a primary enabling technology for Industry 4.0 (AI-based factory automation).

A variety of deep learning-driven techniques have been introduced for acoustic anomaly detection (AAD) in recent years, including dense autoencoders [1,2], convolutional autoencoders [2], and pre-trained convolutional neural networks [3]. Although deep learning-driven methods have demonstrated excellent accuracy in detecting anomalous sounds, the widespread adoption of these methods remains limited. One of the primary reasons for the slow adoption is the prohibitive computational resources required by many deep learning-driven anomaly detection methods, which often have high architectural and computational complexities, as well as high memory footprints. This results in methods that cannot be deployed on resource-constrained edge computing devices such as CPUs or microcontrollers. Moreover, since such methods are typically designed without resource constraints, the on-device prediction latency is typically not an important design parameter. However, for industry deployment, AAD must exhibit real-time latency to be successful, as a quick shutdown of an abnormal sounding machine could be crucial for safety.

Motivated by this challenge, we explore a machine-driven design exploration strategy to create optimal macro-architecture and micro-architecture designs tailored specifically for on-device AAD tasks. Through this strategy, we introduce OutlierNets, a set of highly compact deep convolutional autoencoder network architectures optimized for memory-constrained devices as well as applications where low-latency inference is a key requirement. OutlierNets provide high architectural and computational efficiency while providing high unsupervised AAD accuracy. In addition to assessing *area under the receiver operating curve* (AUC) performance, we further assess the on-device latency of OutlierNet architectures on a variety of devices. These devices include an ARM Cortex A-72 embedded CPU and a desktop class Intel Core i5-7600 K CPU. In the case of the Intel CPU, we leverage domain-specific compilation in the form of the OpenVINO platform for optimal execution.

The remainder of this study as organized as follows. Section 2 provides an overview of existing acoustic anomaly techniques. In Section 3 we discuss the human-driven prototyping, as well as the machine-driven parameter exploration. In Section 3, we include a description of the dataset, mel-spectrograms and the evaluation strategy for the architectures. Section 4 presents architecture results, including parameter count, FLOPs, size in memory, on-device latency and finally the AUC of each network architecture. We finally draw conclusions and discuss potential future directions in Section 5.

## 2. Related Work

A significant amount of attention has been devoted to acoustic anomaly detection over the past decade [4,5,6,7]. Classical techniques such as One-Class Support Vector Machines (OC-SVM) and Gaussian Mixture Models in particular, have been the most popular algorithms in this area. However, classical machine learning techniques generally require feature extraction as an independent step prior to training or inference. For example, Aurino et al. (2014) train OC-SVMs with different types of audio features such as number of zero crossings, auto correlation and Mel-Frequency Cepstral Coefficients (MFCC) to compare their efficacy in detecting anomalous events such as gun-shots and broken glasses. However, such feature extraction can potentially miss the subtle differences between ambient and anomalous sound. In contrast, deep learning based acoustic anomaly detection or deep anomaly detection enables end-to-end learning with automatic extraction of discriminatory features from the audio directly or alternatively from spectrograms.

Until recently, the area of deep learning-driven AAD has been relatively underrepresented in the research literature. This has primarily been due to the lack of publicly available datasets. However, this situation has improved significantly in recent years with the release of AAD datasets such as MIMII [1], and ToyADMOS [8]. Traditionally, autoencoders have been the cornerstone for anomaly detection [2,9,10,11]. In the context of AAD, autoencoders are typically trained in an unsupervised manner on the normal operating sounds of machines. The key idea here is that an autoencoder will learn to reconstruct a machine’s normal sounds with the minimum possible reconstruction error. If the autoencoder is fed with an unseen (anomalous) sound, the reconstruction error would be significantly high, leading to the detection of anomalous events. As a result, the reconstruction error of anomalous sounds would have a different distribution than for normal or ambient sounds.

Autoencoders are typically trained on the features extracted from raw audio signals, with spectral features such as MFCCs [2] and Mel-spectrograms [2,9,10] amongst the most popular. A Mel-spectogram is similar to a conventional spectogram, with the major difference being that the sound is represented on the Mel-scale, which measures the pitch as perceived by humans. The advantage offered by a Mel-spectrogram is that it transforms the time-series representation of the acoustic signal into a rich higher-dimensional representation of frequency, time, and intensity (power), which makes them well-suited for convolutional autoencoder architectures.

Muller et al. [3] proposed the use of ImageNet pre-trained CNNs (e.g., ResNet-18 [12] and AlexNet [13]) for automatic feature extraction from Mel-spectrograms. The extracted features are subsequently fed to traditional machine learning methods such as Gaussian Mixture Models and Support Vector Machines for inference. Although the aforementioned deep learning-driven methods have demonstrated excellent accuracy, the resulting systems require a large amount of computational and memory resources to operate. For example, a ResNet-18 based feature extractor would require 1.8 billion FLOPs for inference and exhibits a memory footprint of over 500 MB. To address the high complexity of such pre-trained CNN architectures, Ribeiro et al. [2] proposed a tailored deep convolutional autonencoder architecture for AAD with a much lower architectural complexity at 4M parameters and a memory footprint of 15 MB. Banbury et al. [14] leveraged a differentiable neural architecture search strategy [15] to design neural network architectures for microcontrollers, the smallest of which is a dense autoencoder architecture with an impressive size of 270 KB and the AUC of 84.7%.

## 3. Methods

In this study, we present OutlierNets, a family of highly compact deep convolutional autoencoder architectures tailored for real-time detection of acoustic anomalies. We include two variants of OutlierNets, OutlierNets${}_{\alpha }$ and OutlierNets${}_{\beta }$. OutlierNets${}_{\alpha }$ is tailored for memory constrained devices whereas OutlierNets${}_{\beta }$ is intended for low latency applications where speed of inference is a priority. These networks were designed with a machine-driven design exploration strategy which was guided by constraints designed to optimize either memory or latency. Human expertise was leveraged to design operational constraints and initial design prototype while the machine-driven design exploration process traverses the architectural search space for optimal network design.

### 3.1. Dataset

To evaluate the OutlierNet designs, we employ the MIMII dataset [1]. The dataset consists of normal and malfunctioning sounds of industrial fans, valves, sliders, and pumps. In this study, we focus on the slider and fan machine types. Each machine type is comprised of recordings from four different machines. For example, for the slider machine type, recordings of four distinct slider machines are provided. Furthermore, the dataset provides recordings with three distinct levels of SNR, 6 dB, 0 dB and −6 dB. We employs all three SNR levels in this study. This combination of SNR levels, machine IDs and machine types results in 24 independent datasets. The training set for each dataset comprises of normal recordings exclusively, whereas the test set is an even mix of malfunctioning and normal sounds. To quantify our detection accuracy, we use the commonly used area under the receiver operating curve (AUC) metric.

### 3.2. Mel-Spectrograms

**Figure 1:**
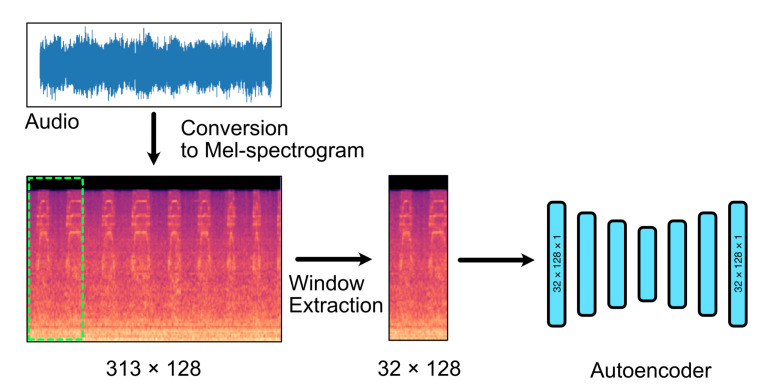
Overview of Mel-spectrogram processing. A given audio signal is transformed to a Mel-spectrogram with 128 Mel bands, hop length of 512 and a Fourier window of 1024. With 10 s of audio, this results in Mel-spectrograms with a size of 313 × 128. Subsequently, we extract nine 32 × 128 pixed sized non-overlapping windows from each Mel-spectrogram. These windows are then fed to an autoencoder for training or for inference. This has the advantage of providing a larger training set and also a smaller image for the autoencoder to encode.

Similar to [2,10], we train OutlierNets on Mel-spectrograms of acoustic recordings rather than time-series representations. Figure 1 provides an overview of Mel-spectrogram processing. Similar to conventional spectrograms, Mel-spectrograms are a time-frequency representation of audio. A conventional spectrogram employs a linear frequency axis to represent the different frequencies in audio. In contrast, a Mel-spectrogram represents the frequency axis in terms of Mel-scales, which mimics human hearing. Humans are able to differentiate lower frequencies with significantly more precision than higher frequencies. For example, the difference between a 250 Hz and a 750 Hz tone will be quite apparent to a typical listener. However, the difference between a 10 kHz and a 10.5 kHz signal will be much more difficult to perceive. The Mel-scale was designed such that equal distances in pitch, sounded equally distant to the typical listener. To transform the linear frequency axis to the Mel-scale, the following expression is employed

$$m=2595\log_{10}\left(1+\frac{f}{700}\right)$$

where *m* is the mel band and *f* is the frequency to be mapped. We employ 128 Mel bands with a hop length of 512, with the Fourier window set to 1024, resulting in 313×128 Mel-spectrograms. The resulting Mel-spectrogram is then cropped into 32×128 windows with no overlap. Each window represents approximately one second of audio. This is crucial for two reasons. First, shorter windows would extract a relatively homogeneous part of the Mel-spectrogram, making it easier for the autoencoder to learn the latent representation. Second, a shorter window facilitates the proposed system to detect anomalous sound with only one second of recorded audio. This is an important consideration for real-time AAD. A system that requires a considerably longer recording would have an inherent lag built into the system and would not be real-time, even if the prediction itself is rapid.

An example of a Mel-spectrogram is given in Figure 1 along with the cropping boxes. Note that the small window would not deteriorate the autoencoder’s ability to detect anomalous sound, even if the duration of the acoustic anomaly is longer than one second. This is because if an acoustic anomaly completely encompasses a 32 × 128 window, the autoencoder would still exhibit a substantially high reconstruction error, assuming that the anomalous sound is different from the normal operating sounds of the machine.

### 3.3. Network Architecture Prototyping

The first step in the human-machine collaborative design strategy involves implementing a prototype network that is subsequently optimized via generative synthesis. Our initial experiments showed that anomaly detection for the slider machine was more difficult than for the fan. Keeping this in mind, we implement two distinct convolutional autoencoders as prototypes, with the one intended for the slider machine featuring an extra convolution layer in the encoder and decoder. Both autoencoders are fed with the 32×128 Mel-spectrogram window without any additional preprocessing. Additionally, both models use a 3×3 convolutional kernel. Our initial experiments also showed that despite the relatively shallow depth of the autoencoders, the results benefited from the use of batch normalization after each convolutional layer.

The larger autoencoder features approximately 133,000 parameters and 5 MFLOPs. The encoder comprises four convolutional layers with filter banks of 4, 8, 16, and 32. The latent space features a dense layer with 40 nodes. The decoder is a mirror of the encoder, yielding 8 convolutional layers and 1 dense layer. The smaller network has an encoder with three convolutional layers with filter banks of 8, 16, and 32. The decoder, again, is a mirrored version of the encoder with transposed convolution. This smaller autoencoder exhibits approximately 20,000 parameters.

### 3.4. Machine-Driven Design Exploration

Prior to advances in machine-driven exploration of architectural search spaces, the design of neural network architectures was a very challenging and time-consuming process. Moreover, the required resource constraints necessary for real-time edge scenarios (e.g., architectural and computational complexities, memory footprint, etc.) makes this process even more challenging. Given the number of design hyperparameters involved, the architectural search space of even simple neural networks is often extremely large. This led to the great recent interest in machine-driven methods for exploring the architectural search space to find the optimal network given a set of constraints or design objectives.

The problem of traversing the architectural search space for an optimal neural network can be generally framed in two different ways. The first category of approaches [14,16,17] formulates the search as a multi-objective optimization problem. The objectives can include criteria such as network size, inference latency, accuracy or FLOPs. In these approaches, optimization is performed via reinforcement learning, evolutionary algorithms, or gradient descent. The second category of approaches [18] leverages a constrained optimization problem formulation, which consists of an objective function (e.g., [19]) as well as a set of operational constraints such as accuracy, computational and architectural requirements.

In this investigation, we leverage the second approach via generative synthesis [18] to automatically explore the architecture design space and obtain optimal network designs for on-device real-time AAD. Generative synthesis can be formulated as a constrained optimization problem, where the goal is to obtain a tailored deep neural network architecture as determined by an optimal generator G which, given seeds S, generates neural network architectures that maximize a performance function U while satisfying the constraints defined in an indicator function $1_{r}\left(\cdot \right)$,

(1) $$G=\max_{G}U\left(G\left(s\right)\right)\;\;\;\mathrm{subject}\mathrm{to}\;\;\;1_{r}\left(G\left(s\right)\right)=1,\;\;\forall \in S.$$

Given an initial design prototype φ, U, and $1_{r}\left(\cdot \right)$, the approximate solution to this constrained optimization problem can be found in an iterative fashion. We leverage human expertise and define a convolutional autoencoder design as the initial prototype φ. The prototype has an input of a 32×128 pixel Mel-spectrogram, with convolutional kernel sizes defined as 3×3. Since this study aims to design architectures optimized for memory and latency, we employ two indicator functions, $1_{r}^{\alpha }$ and $1_{r}^{\beta }$, both of which impose constraints on parameter count and AUC to yield efficient and accurate designs. We refer to architectures obtained via $1_{r}^{\alpha }$ and $1_{r}^{\beta }$ as OutlierNets${}_{\alpha }$ and OutlierNets${}_{\beta }$, respectively. Recall that OutlierNets${}_{\alpha }$ is optimized for memory footprint whereas OutlierNets${}_{\beta }$ is optimized for low latency. To this end, $1_{r}^{\beta }$ prohibits the use of depthwise convolution and forces OutlierNets${}_{\beta }$ to rely solely on standard convolution. Although depthwise convolution typically requires far fewer FLOPS and parameters, it also exhibits poor arithmetic intensity (ratio of computation to memory access) and is typically memory bound when compared with standard convolution. The poor arithmetic intensity results in potentially worse real world architectural latency in hardware architectures that do not fully accelerate depthwise convolution. Therefore, prohibiting depthwise convolution in OutlierNets${}_{\beta }$ results in faster, lower latency architectures whereas allowing depthwise convolutions in OutlierNets${}_{\alpha }$ yields in memory and storage efficient architectures.

**Figure 2:**
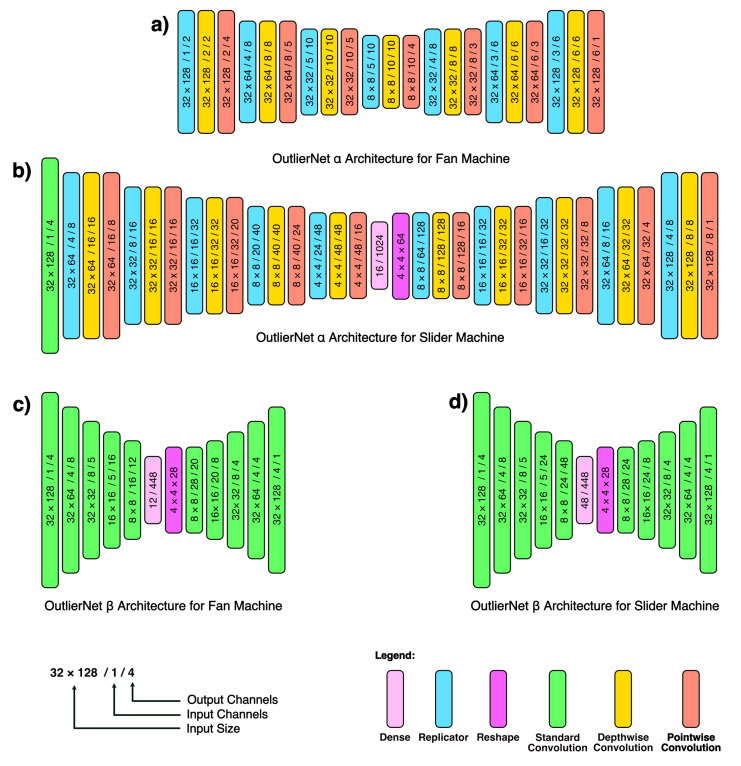
(**a**) Example of an OutlierNets${}_{\alpha }$ autoencoder architecture for fan AAD tasks (ID: 06, SNR: 6 dB) (**b**) Example of an OutlierNets${}_{\alpha }$ autoencoder architecture for slider AAD tasks (ID: 00, SNR: −6 dB). (**c**) Example of OutlierNets${}_{\beta }$ID: 02, SNR: 0 dB architecture for fan AAD tasks (ID: 04, SNR: 6 dB). (**d**) Example of OutlierNets${}_{\beta }$ architecture for slider AAD tasks (ID: 04, SNR: 6 dB).

We emphasize that the generative-synthesis process is entirely automatic, yielding an architecture during each step of the iteration which is evaluated for a given performance metric (e.g., validation accuracy, AUC etc.), parameter count and FLOPS. The final selection network is based upon these metrics. Examples of the final OutlierNets${}_{\alpha }$ and OutlierNets${}_{\beta }$ architectures are shown in Figure 2. A number of observations can be made about the designed OutlierNet architectures. First, the designed OutlierNets${}_{\alpha }$ possess very lightweight macro-architecture comprised largely of depthwise convolutions, pointwise convolutions, and replicators, all contributing to a low parameter count network architecture. Second, the designed OutlierNet architectures exhibit high micro-architecture design diversity to further reinforce a strong balance between AAD accuracy and efficiency. Third, the OutlierNet architectures exhibit macro-architecture differences between architectures tailored for fan AAD tasks and architectures for slider AAD tasks, with architecture designs for slider ADD tasks being more complex (e.g., additional standard convolution layers and a dense latent space) given that slider ADD is a tougher task. Finally, we can note that the imposition of $1_{r}^{\beta }$ yields network architectures with only standard convolution (Figure 2c,d).

### 3.5. Deployment Hardware

To assess real world performance, this study deploys OutlierNet architectures on two different devices, a 3.8 GHz Intel Core i5-7600 K CPU and an ARM Cortex A72 embedded CPU. To establish a baseline for our performance, we deploy CPU-accelerated models on the desktop class Intel chip. The OutlierNet network architectures are passed through a domain-specific compiler built for Intel CPUs (i.e., OpenVINO [20]) for CPU-accelerated latency experiments. This compiler optimizes the inference in two key ways. The first is that it optimizes cache access such that the CPU does not have to fetch data continuously from the much slower main memory. This alone yields significant gains in efficiency. Additionally, OpenVINO optimizes the network graph such that sequential operations, like convolution and subsequent activation, are fused together. This domain-specific compilation thus yields CPU-accelerated models that feature a significantly lower inference latency and demonstrate efficiency of the proposed OutlierNet architectures.

Additionally, in order to test performance on devices that are likely to be deployed in industry for machine learning tasks, we employ an ARM Cortex A72 with 4 GB of RAM. The ARM Cortex A72 was chosen due to its widespread usage in industry already.

## 4. Results and Discussion

To evaluate the efficacy of the OutlierNet architecture designs, we compare their AUC, parameter counts, model sizes, FLOPs, and on-device latencies with the state-of-the-art deep convolutional autoencoder architecture introduced in [2]. Similar to our approach, [2] employ small overlapping Mel-spectrograms to classify anomalous sounds. For brevity, we refer to their proposed architecture as **CAE-MCS** as a shorthand for **c**onvolutional **a**utoencoder for **m**achine **c**ondition **s**ounds for the remainder of this study. To provide a fair comparison, we implement and evaluate CAE-MCS on the fan and slider datasets from the MIMII dataset. We found this architecture to achieve high AAD performance, reporting an average AUC of 89.1% for slider AAD tasks and 83.5% for fan AAD tasks while only having 4 M parameters. As an additional experiment, we scale down the CAE-MCS architecture to create a more efficient architecture (which we will refer to as **CAE-MCS-S**) such that it features a similar number of parameters (72,807) as the largest OutlierNet network (69,877). Similar to CAE-MCS, we train the smaller CAE-MCS-S model on the fan and slider datasets to evaluate if scaling down a state-of-the-art architecture is sufficient to yield an optimal balance between efficiency and accuracy. The remainder of this section provides in-depth assessment of the architectural complexity (Table 1), model performance (Table 2) and overall detection accuracy (AUC) of the found architectures (Table 3).

Table 1 lists the parameter count and model size of OutlierNets${}_{\alpha }$, OutlierNets${}_{\beta }$, CAE-MCS and CAE-MCS-S. We observe that the proposed OutlierNets${}_{\alpha }$ architectures exhibit very diverse architecture and computational complexities for each of the 24 AAD tasks tested in this study, all tailored around the complexity of the task at hand. Specifically, CAE-MCS has a parameter count of approximately 4 million, whereas the smallest and largest OutlierNets in comparison have 686 parameters **(5800× fewer)** and 70,000 parameters **(57× fewer)**. The CAE-MCS architecture translates to a model size of ∼15 MB, which may be impractical to deploy for certain on-device edge scenarios, particularly those leveraging microcontrollers. In contrast, the smallest OutlierNet (at **2.7 KB**) can fit within the Static RAM of most microcontrollers whereas the larger OutlierNets are within reach of most higher-end microcontrollers such as the STM32F7 series. Here we can see the clear advantage of allowing depthwise convolutions in OutlierNets${}_{\alpha }$ via the $1_{r}^{\alpha }$ indicator function, yielding extremely small architectures. In contrast, the $1_{r}^{\beta }$ indicator function yields comparatively larger architectures but still significantly smaller than CAE-MCS and CAE-MCS-S. Specifically, we note that the smallest OutlierNets${}_{\beta }$ architecture features 12,533 parameters, approximately **320×** fewer than CAE-MCS and **5.76×** fewer than CAE-MCS-S.

Next, we investigate the on-device latency of OutlierNets, CAE-MCS-S and CAE-MCS on a Intel Core i5-7600K CPU and an ARM Cortex A72 embedded CPU (Table 2). The details of these devices are described in Section 3.5. To assess the performance of OutlierNets, we measure the latency of each architecture as well as CAE-MCS and CAE-MCS-S on each of the aforementioned devices. We employ a batch size of 1 for each measurement and close all user processes to ensure minimal CPU overhead. It is important to note that any error in the measurements, either due to background processes or even thermal throttling, would increase the latency (i.e., the error is positive). Additionally, background processes and thermal throttling would introduce some variable in measurements. To mitigate this inconsistency in measurements, we record the latency of each architecture one hundred times and report the minimum time recorded. These results are shown in Table 2 which compares the on-device latency between OutlierNets${}_{\alpha }$, OutlierNets${}_{\beta }$, CAE-MCS-S and CAE-MCS.

The CPU-accelerated latency experiments on an Intel Core i5-7600K showed that CAE-MCS-S and CAE-MCS achieved an on-chip runtime latency of 1.1 μ and 7.7 μs, respectively. The fastest and slowest OutlierNets${}_{\alpha }$ in comparison achieved an on-chip latency of 0.366 μs **(21 × faster)** and 0.746 μs **(∼10.3 × faster)**, respectively. This difference in inference latency holds even when the models are deployed on an ARM Cortex A-72, a significantly slower and power constrained device when compared to the desktop class Intel Core i5. Specifically, we note that the CAE-MCS model now has an on-device latency of 78 µs while the fastest and slowest OutlierNets${}_{\alpha }$ achieve 7.2 µs (**10.8 × faster**) and 19.1 µs (**∼4.1 × faster**). The fastest OutlierNets${}_{\alpha }$, in contrast, reports a latency of 6.044 μs (**5 × faster**) while the slowest exhibits a latency of 13.575 μs (**2.33 × faster**). Next, we observe the benefit of prohibiting depthwise convolution by the $1_{r}^{\beta }$ indicator function. We note that, on average, OutlierNets${}_{\beta }$ are twice as fast as OutlierNets${}_{\alpha }$ on the Intel CPU and as well the ARM Cortex A72. As explained in Section 3.4, this difference in performance is due to the arithmetic density of standard convolution over depthwise convolution. This improvement in performance enables OutlierNets${}_{\beta }$ to be **30×** faster than CAE-MCS and **4.4×** faster than CAE-MCS-S.

Finally, in Table 3 we compare the AUC of OutlierNets with CAE-MCS and CAE-MCS-S. Most importantly, we observe that there is almost no difference between the AUC of OutlierNets${}_{\alpha }$ and OutlierNets${}_{\beta }$, despite the difference in model sizes. This is due to the machine-driven exploration of the architectural search space, enabling an optimal balance to be found between the AUC and the latency/memory-footprint. Secondly, it can be observed that the proposed OutlierNet architectures achieved AUC scores matching or exceeding the much larger CAE-MCS deep convolutional autoencoder architecture for both the slider and the fan datasets despite being orders of magnitude lower in architectural and computational complexities. In particular, OutlierNets achieve **88.8%** average AUC, across all slider machines and SNRs, whereas CAE-MCS achieves 89.1%. Similarly, the proposed OutlierNet architectures achieve **83.0%** average AUC for fans while CAE-MCS achieves 83.5%. Perhaps most importantly, we note that CAE-MCS-S architecture has an average AUC of 0.84, approximately 3% lower than OutlierNets. This demonstrates that simply scaling down a larger architecture does not yield an accurate or efficient model. In contrast, when the network architecture is tailored for a specific task (e.g., to optimize AUC and latency), we observe improvements in AUC, model size and latency as the tailored architecture strikes an optimal balance between AUC and memory or latency. Notably in some experiments for the slider task (ID:00-04, SNR:6db), OutlierNets${}_{\beta }$ are able to provide smaller memory footpring and a lower latency than OutlierNets${}_{\alpha }$.

The aforementioned results demonstrate the efficacy of a machine-driven design exploration strategy in constructing deep neural network architectures that strikes a strong balance between accuracy and efficiency. The resulting OutlierNet architectures achieved AUC scores matching or exceeding the much larger CAE-MCS deep convolutional autoencoder architecture for both the slider and the fan datasets despite being orders of magnitude lower in architectural and computational complexities. The resulting architectures are simple enough to fit within the SRAM of most microcontrollers while exhibiting real-time on-device latency. The low memory requirements and the demonstrated microsecond level on-chip latency on embedded hardware results in systems that can be deployed on factory floors for automated AAD.

## 5. Conclusions

We explored a machine-driven design exploration approach that leverages both human experience and knowledge with the speed and meticulousness of a machine to produce highly compact deep convolutional autoencoder architectures tailored for the purpose of AAD on embedded devices. The resulting OutlierNets possess parameter counts ranging from 686 to 69,877, which translate to model sizes of 2.7 KB to 273 KB. With such low resource requirements, these models can fit within the SRAM of many microcontrollers available today. Despite extremely low complexities, OutlierNets match or exceed the AUC of a much larger convolutional autoencoder architecture while exhibiting microsecond scale latency on embedded hardware. As future work, we aim to study the types of architectures that can be created using machine-driven design exploration with other machine operating sound types, and study design exploration considerations tailored for even lower-power microcontrollers to facilitate compact and real-time AAD on edge devices.

## Figures and Tables

**Table 1:** Size comparison between OutlierNet architectures with the convolutional autoencoder architecture introduced in [2] (CAE-MCS) as well as a reduced baseline (CAE-MCS-S) in terms of parameter count and memory footprint.

Type	SNR	ID	OutlierNets${}_{\alpha }$		OutlierNets${}_{\beta }$		CAE-MCS-S	CAE-MCS
			Params	Size (KB)	Params	Size (KB)		
Fan Machine	6 dB	00	2028	7.9	12,533	49.0	72,252 Params 282.2 KB	4M Params 15 MB
		02	907	3.5	13,391	52.3		
		04	3055	11.9	12,620	49.3		
		06	686	2.7	13,631	53.2		
	0 dB	00	2992	11.7	13,699	53.5		
		02	1067	4.2	64,856	253.3		
		04	1025	4.0	21,235	282.2		
		06	861	3.4	16,491	64.4		
	−6 dB	00	6840	26.7	64,856	253.3		
		02	2892	11.3	14,404	56.3		
		04	1826	7.1	12,546	49.0		
		06	2919	11.4	72,252	282.2		
Slider Machine	6 dB	00	21,781	85.1	12,427	48.5		
		02	69,877	273.0	20,960	81.9		
		04	58,729	229.4	21,755	85.0		
		06	53,705	209.8	44,620	174.3		
	0 dB	00	21,781	85.1	25,006	97.7		
		02	69,877	273.0	65,211	254.7		
		04	42,377	165.5	37,652	147.1		
		06	58,157	227.2	65,016	254.0		
	−6 dB	00	27,229	106.4	38,007	148.6		
		02	27,229	106.4	43,578	170.2		
		04	57,497	224.6	65,271	254.9		
		06	58,157	227.2	65,016	254.0		
	Average		24,729	96.6	31,784	124.2		

**Table 2:** Efficiency comparison between OutlierNet architectures with the convolutional autoencoder architecture in [2] in terms of FLOPS and on-device latency with an ARM Cortex-A72 embedded CPU and an Intel Core i5-7600K desktop CPU. These measurements are included in the columns *ARM (μs)* and *Intel (μs)*.

Machine	SNR	ID	OutlierNets${}_{\alpha }$			OutlierNets${}_{\beta }$			CAE-MCS-S	CAE-MCS
			FLOPS	ARM (µs)	Intel (µs)	FLOPS	ARM (µs)	Intel (µs)		
Fan	6 dB	00	7.3 M	11.16	0.53	1.8 M	6.51	0.26	6.5 µs ARM 1.1 µs Intel	78.2 µs ARM 7.7 µs Intel
		02	3.6 M	7.16	0.38	1.8 M	6.62	0.28		
		04	10.6 M	12.45	0.62	1.7 M	6.65	0.28		
		06	2.8 M	7.60	0.37	1.9 M	6.48	0.28		
	0 dB	00	10.3 M	18.15	0.57	1.8 M	6.68	0.28		
		02	4.1 M	12.31	0.41	2.0 M	6.92	0.28		
		04	4.1 M	12.78	0.43	2.4 M	6.97	0.28		
		06	3.3 M	12.85	0.38	1.9 M	6.67	0.27		
	−6 dB	00	22.9 M	15.64	0.75	2.0 M	6.64	0.28		
		02	10.5 M	19.07	0.62	1.9 M	6.68	0.26		
		04	6.4 M	16.37	0.48	1.7 M	6.72	0.28		
		06	10.1 M	17.72	0.58	2.4 M	6.87	0.28		
Slider	6 dB	00	4.6 M	10.51	0.47	1.7 M	6.67	0.26		
		02	5.3 M	10.99	0.50	1.7 M	6.67	0.28		
		04	5.1 M	10.84	0.49	1.8 M	6.64	0.28		
		06	5.1 M	10.89	0.49	1.9 M	6.51	0.28		
	0 dB	00	4.6 M	10.57	0.46	1.8 M	8.93	0.28		
		02	5.3 M	10.93	0.50	1.9 M	6.44	0.28		
		04	4.9 M	10.60	0.47	1.9M	6.52	0.26		
		06	5.2 M	10.76	0.49	2.0 M	6.68	0.28		
	−6 dB	00	4.7 M	10.73	0.48	1.8 M	6.73	0.27		
		02	4.6 M	10.73	0.46	1.9 M	6.81	0.28		
		04	5.1 M	10.75	0.48	2.0 M	6.62	0.28		
		06	5.1 M	10.90	0.49	2.0 M	6.71	0.28		

**Table 3:** Anomaly detection accuracy (AUC) comparison between OutlierNets${}_{\alpha }$, OutlierNets${}_{\beta }$ architectures, CAE-MCS-S and CAE-MCS [2]. The models are evaluated on the Fan Machine and Slider Machine from the MIMII Dataset.

Type	SNR	ID	OutlierNets${}_{\alpha }$	OutlierNets${}_{\beta }$	CAE-MCS-S	CAE-MCS
Fan	6 dB	00	0.87	0.84	0.76	0.84
		02	0.99	0.99	0.99	0.99
		04	0.97	0.94	0.88	0.94
		06	1.00	1.00	1.00	1.00
	0 dB	00	0.62	0.63	0.57	0.63
		02	0.92	0.93	0.89	0.93
		04	0.80	0.79	0.78	0.82
		06	1.00	1.00	0.99	0.99
	−6 dB	00	0.52	0.53	0.51	0.55
		02	0.71	0.73	0.75	0.77
		04	0.61	0.60	0.60	0.64
		06	0.97	0.95	0.95	0.93
	Average		0.83	0.83	0.81	0.83
Slider	6 dB	00	0.99	1.00	0.99	1.00
		02	0.97	0.95	0.97	1.00
		04	0.98	0.96	0.96	1.00
		06	0.91	0.90	0.87	0.90
	0 dB	00	0.99	0.99	0.99	0.99
		02	0.88	0.85	0.85	0.93
		04	0.94	0.88	0.91	0.93
		06	0.75	0.71	0.68	0.73
	−6 dB	00	0.96	0.97	0.94	0.96
		02	0.79	0.76	0.78	0.81
		04	0.87	0.88	0.83	0.85
		06	0.62	0.60	0.59	0.64
	Average		0.89	0.87	0.86	0.89

## Data Availability

Publicly available datasets were analyzed in this study. This data can be found at https://zenodo.org/record/3384388, accessed on 13 July 2021.
